# Radiological and pathological size estimations of pure ductal carcinoma *in situ* of the breast, specimen handling and the influence on the success of breast conservation surgery: a review of 2564 cases from the Sloane Project

**DOI:** 10.1038/sj.bjc.6605513

**Published:** 2010-01-05

**Authors:** J Thomas, A Evans, J Macartney, S E Pinder, A Hanby, I Ellis, O Kearins, T Roberts, K Clements, G Lawrence, H Bishop

**Affiliations:** 1 Pathology Department, Western General Hospital and Breakthrough Breast Cancer Research Unit, Edinburgh EH4 2XU, UK; 2 Division of Medical Sciences, Centre for Oncology and Molecular Medicine, University of Dundee, Ninewells Hospital, Dundee DD1 9SY; 3 Pathology Department, University Hospitals Coventry and Warwickshire, Coventry, CV2 2DX, UK; 4 Research Oncology, Division of Cancer Studies, King's College London, London, SE1 9RT, UK; 5 Section of Pathology and Tumour Biology, Yorkshire Cancer Research and Liz Dawn Pathology and Translational Science Centre, Leeds Institute of Molecular Medicine, Wellcome Trust Brenner Building, St James’ University Hospital, Beckett Street, Leeds, LS9 7TF, UK; 6 Pathology Department, City Hospital, Nottingham, NG5 1PB, UK; 7 West Midlands Cancer Intelligence Unit, University of Birmingham, Birmingham, B15 2TT, UK; 8 Breast Unit, Royal Bolton Hospital, Bolton, BL4 0RJ, UK

**Keywords:** DCIS measurement, pathology, radiology, breast screening

## Abstract

**Background:**

The Sloane Project, an audit of UK screen-detected non-invasive carcinomas and atypical hyperplasias of the breast, has accrued over 5000 cases in 5 years; with paired radiological and pathological data for 2564 ductal carcinoma *in situ* (DCIS) cases at the point of this analysis. We have compared the radiological estimate of DCIS size with the pathological estimate of DCIS size. We have correlated these sizes with histological grade, specimen-handling methods, particularly the use of specimen slice radiographs, and the success or failure of breast-conserving surgery (BCS).

**Methods:**

The Sloane Project database was interrogated to extract information on all patients diagnosed with DCIS with complete radiological and pathological data on the size of DCIS, nuclear grade, specimen handling (with particular reference to specimen radiographs) and whether primary BCS was successful or whether the patient required further conservation surgery or a mastectomy.

**Results:**

Of 2564 patients in the study, 2013 (79%) had attempted BCS and 1430 (71%) had a successful single operation. Of the 583 BCS patients who required further surgery, 65% had successful conservation and 97% of them after a single further operation. In successful one-operation BCS patients, there was a close agreement between radiological and pathological DCIS size with radiology tending to marginally overestimate the disease extent. In multiple-operation BCS, radiology underestimated DCIS size in 59% of cases. The agreement between pathological and radiological size of DCIS was poor in mastectomies but was improved by specimen slice radiography, suggesting specimen-handling techniques as a cause.

**Conclusion:**

In 30% of patients undergoing BCS for DCIS, preoperative imaging underestimates the extent of disease resulting in a requirement for further surgery. This has implications for the further improvement of preoperative imaging and non-operative diagnosis of DCIS so that second operations are reduced to a minimum.

The clinical management of ductal carcinoma *in situ* (DCIS) of the breast remains problematic. The disease is thought to spread radially along the duct systems in the breast (Faverly *et al*, 1994; Vicini *et al*, 2001), and treatment demands a close cooperation between surgeons, radiologists and pathologists if the true size of the disease can be accurately assessed preoperatively. The radiologist will identify the mammographic abnormality, but crucially must help the surgeon by accurate measurement. The surgeon must meticulously orientate the surgical specimen to help the pathologist define the margins. It has been previously shown that there is a correlation between size of disease and the requirement for further surgery, underlining the need for accurate preoperative assessment (Vicini *et al*, 2001).

The Sloane Project (The Sloane Project, 2009), named in memory of the late Professor John Sloane, is a prospective UK audit of patients with screen-detected non-invasive carcinomas and atypical hyperplasias of the breast detected by the National Health Service Breast Screening Programme (NHSBSP). The audit started in 2003 with the aim of assessing the effect of current clinical management on the long-term outcome of patients. All the UK NHSBSP breast-screening units are encouraged to participate in the project, and 77 out of 95 (81%) of the UK screening units voluntarily submit the data. Each clinical specialty in a screening unit contributes profession-specific data relating to diagnosis and treatment using specially designed data collection forms. The data recorded include details of specimen radiology and orientation, the radiological and pathological size, and surgical margins. There is published guidance for the UK Breast Screening Programme on the optimal standards for specimen handling of the resected DCIS (Ellis *et al*, 2005). Further guidance is also available from the Sloane Project website (The Sloane Project, 2009).

The key problem in treating patients with DCIS is tumour-positive surgical margins after breast-conserving surgery (BCS), which is likely to be associated with residual DCIS and a high risk of local recurrence (Dunne *et al*, 2009). Prevention of local recurrence is the main issue in treating patients with a curable *in situ* carcinoma as half of these recurrences are invasive carcinomas (Silverstein *et al*, 1998). This underlines the value of accurate preoperative measurement to guide BCS.

This paper examines specifically the relationship between radiological and pathological size measurements, their relationship to DCIS grade, specimen-handling techniques (particularly specimen slice radiography) and whether BCS was successful as a primary procedure.

## Materials and methods

The period of study covered all entrants to the Sloane Project from its inception in April 2003–December 2008 – over 5000 patients. All patients were screen-detected with an age range of 50–70 years and a screening interval of 3 years. Approximately 70% of the entered patients had either a missing pathological or radiological data form at the time of analysis with some overlap giving an initial study population of 3883 patients who had both pathological and radiological data forms. In total, a further1319 cases were excluded from the study for the following reasons: unpaired pathological/radiological measurements (644), atypical ductal hyperplasia and/or lobular *in situ* neoplasia alone or in combination with DCIS (304), inappropriate first operation recorded (e.g., axillary surgery only, therapeutic re-excision) (39), DCIS grade not recorded (28) or if a diagnostic biopsy was performed (304) and thus true pathological size was not assessable. There was an overlap between these groups. The 304 diagnostic biopsies with paired size data and in which DCIS grade was recorded were excluded from the main study but will be analysed briefly in the Results section.

The remaining 2564 cases on the database were interrogated to identify three groups of patients with pure DCIS who had either a single successful breast-conserving operation (*n*=1430), a mastectomy as a primary procedure (*n*=551) or further surgery (re-excision and/or completion mastectomy) because of failed primary BCS the first time around (*n*=583). These three groups were compared for radiological and pathological size agreement, DCIS grade and whether specimen slice radiography had been carried out. These groups were chosen because they allowed a clear comparison of factors associated with the success or failure of BCS and also case selection for mastectomy.

We do not have precise records of imaging technique but we estimate that >90% of patients will have received film-screen mammography with only a small percentage receiving computerised radiography or digital mammography. The Sloane Project radiology form enables bi-dimensional recording of the disease extent. The radiological size is the largest diameter on either the craniocaudal or oblique view. This measurement is taken after viewing the magnification views to look for subtle additional calcifications, but not usually directly measured from the magnification views. A minority of cases will have had full-field digital mammography at assessment and in these cases the measurements will have been done from electronic magnification of the standard views on a digital workstation. Measurements were not taken from specimen radiographs. Intraoperative radiographs were read by the surgeon but had no influence on the final determination of radiological size, which is a preoperative measurement. For the purposes of this study, the maximum recorded radiological size of either the length or the diameter of the lesion was used.

The pathological measurement of DCIS size includes both the primary excision and any additional disease found at subsequent re-excision or mastectomy. Screen-detected DCIS in the United Kingdom is graded by nuclear morphology alone, as guided by the NHSBSP Reporting Guidelines (Ellis *et al*, 2005).

### Data analysis

Data were analysed using ‘Analyse it’ version 2.20 software for Microsoft Excel. Measurements of agreement between radiological and pathological sizes were assessed using Altman–Bland plots to compare the difference between two measurement techniques of a continuous variable (pathology and radiology in this study) (Altman and Bland, 1983; Bland and Altman, 1986). The differences between the two paired measurements are plotted against the mean of values obtained using the two techniques. The mean of the differences is calculated to give a measure of ‘bias’ of one technique over the other. This may be a positive or negative value. The differences between radiological and pathological paired measurements for the various subgroups were compared using the Mann–Whitney *U*-test. Comparisons of frequencies were made using the *χ*^2^-test. We defined an *α*-error for tests of significance at *P*=0.05.

## Results

### All cases

The distribution of DCIS grade in patients having one successful BCS operation, primary mastectomy and failed primary BCS groups is shown in Table 1. Overall, 67% of cases had high-grade disease, 26% intermediate grade and 7% low grade. Those patients who were offered mastectomy as a primary procedure had a higher incidence of high-grade disease (80%) than those who had successful primary BCS (60%) or unsuccessful primary BCS (71%). These differences between groups for high-grade DCIS are all highly significant (*P*⩽0.0001).

Specimen radiography by the surgeon was carried out in 1811 of 2564 (71%) cases. Overall, specimen slice radiography was carried out by pathologists in only 757 of 2564 (29%) cases. There was no difference in the use of specimen slice radiography between women having successful one-operation BCS or unsuccessful BCS (31 *vs* 32%). Of the one-operation mastectomy specimens, 22% had slice radiography. For all cases, pathologists who elected to X-ray specimen slices took more blocks than those who did not (one-operation BCS: median=12 *vs* 10 per case; one-operation mastectomy: 17 *vs* 13 per case; and two operations or more: 14 *vs* 10 per case). These differences are all highly significant (all *P*⩽0.0001).

Although 445 cases out of the original 3883 had large blocks taken as part of the specimen handling in the study population of 2564 cases, only 10 were subjected to this technique.

### Patients undergoing a single BCS procedure

The agreement between median maximum radiological and pathological DCIS sizes was very close in successful one-operation BCS patients (12 mm *vs* 14 mm) (Table 2). The level of agreement was not affected by specimen slice radiography but was affected by the DCIS grade (overall difference: 2 mm in low-grade *vs* 1 mm in high-grade disease).

### Patients undergoing a mastectomy as a primary procedure

**Figure 1:**
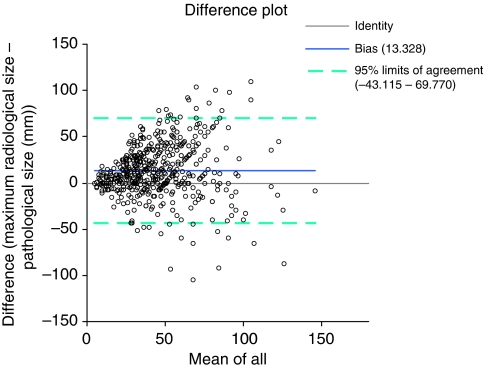
Altman–Bland agreement plot for primary mastectomies. The solid line shows the measure of bias (13.33 mm). The 95% confidence intervals refer to the differences between radiological and pathological measurements and are shown as broken lines. The colour reproduction of the figure is available on the html full text version of the paper.

The agreement between radiological and pathological maximum DCIS size was less good in primary procedure mastectomy specimens with the overall median radiological size being 18 mm larger than the pathological size (50 mm *vs* 32 mm) (Table 2). The difference between radiological and pathological size increased with decreasing grade (15 mm for high-grade DCIS and 46 mm for low-grade disease). The level of agreement between radiological and pathological size was generally improved by specimen slice radiography (difference: 14 mm *vs* 17 mm for all cases) with the exception of low-grade DCIS in which the difference increased from 32 to 62 mm, but case numbers for this group were low. An example of an Altman–Bland plot for the primary mastectomy group is shown in Figure 1.

### Failed primary BCS – patients requiring re-excision or mastectomy

Of the 2013 patients who underwent BCS as a primary procedure, 583 (30%) required further surgery because of involved margins. Two-thirds of these 583 patients ultimately had successful breast conservation, the majority of these after a single further operation. One-third of these patients required mastectomy. These results are summarised in Table 3.

**Figure 2:**
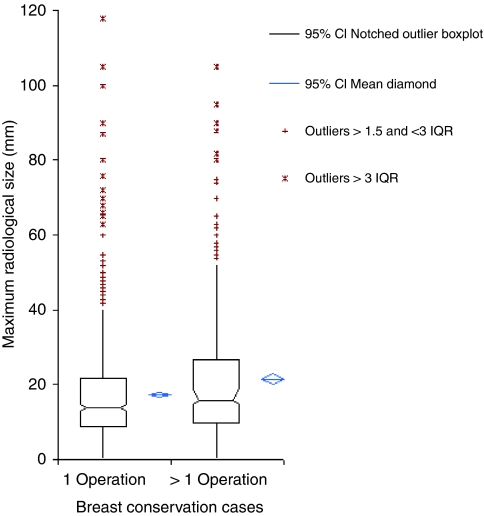
Maximum radiological size distributions for successful (1 operation) *vs* unsuccessful (>1 operation) breast conservation cases. CI, confidence interval; IQR, interquartile range.

In this group of patients, who required subsequent re-excision in the form of additional breast conservation surgery or mastectomy, the radiological size of DCIS was significantly greater than the one-operation BCS group (*P*⩽0.0001). This is true for all histological grades (high grade: *P*=0.002; intermediate grade: *P*=0.03; low grade: *P*=0.02). The distribution of radiological sizes is shown in Figure 2. The radiological size of disease was also substantially less than that demonstrated pathologically (16 mm *vs* 23 mm) (Table 2). This mismatch was exaggerated in low-grade DCIS (15 mm *vs* 27 mm). Specimen slice radiography improved the agreement between radiological and pathological size (overall difference: 4 mm *vs* 7 mm). This effect was particularly marked for low-grade disease (7 mm *vs* 15 mm).

**Figure 3:**
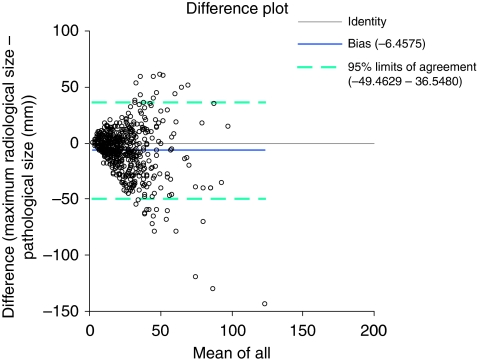
Altman–Bland plot for agreement between radiological and pathological size measurements for all unsuccessful (2+operations) breast-conserving surgery (BCS) cases. Note that the measure of overall bias is now negative (mean: −6.46 mm). The 95% confidence intervals refer to the differences between radiological and pathological measurements and are shown as broken lines.

There was no difference in median specimen weight (55 *vs* 58 g). A record of whether radiological calcification was present or not was made in 2558 out of 2564 (>99%) cases. There was no significant difference between recorded calcification in the two conservation groups (92.0 *vs* 91.6%). We have not carried out a detailed review of the type of calcification recorded in this study. A representative Altman–Bland difference plot from this group is shown in Figure 3 demonstrating the negative measure of bias in this group.

The trend of worsening agreement between radiological and pathological measurement of DCIS from conservation to mastectomy specimens, from high-grade to low-grade disease and from successful single BCS to failed primary BCS is underlined by the increasing Altman–Bland bias from one group to the next (Table 4).

Although the differences between radiological and pathological size at different grades within each group are not significant, the difference between the one-operation BCS (*n*=1430) and the failed primary BCS (*n*=583) groups as suggested by the polarised Altman–Bland plots is highly significant (*P*⩽0.0001). The differences between radiological and pathological sizes comparing the one-operation BCS group and the one-operation mastectomy group are also highly significant (*P*⩽0.0001) as is the difference between radiological and pathological sizes in the one-operation mastectomy and two-operation groups (*P*⩽0.0001).

### Radiological–pathological size differences and the outcome of BCS

**Figure 4:**
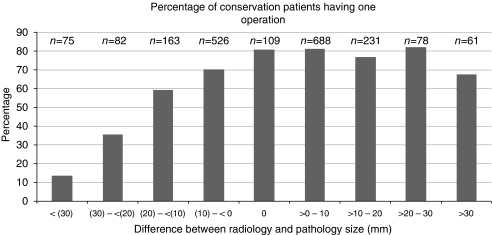
Percentage of all cases treated initially by breast conservation surgery having a single successful operation compared with radiological–pathological size difference in mm. Note that the size differences in parentheses are negative. *n*=numbers of cases in each difference band.

In the one-operation BCS group, 503 of 1430 patients (35%) showed a pathological DCIS size exceeding the radiological size compared with 343 of 583 (59%) in the failed BCS group. In all BCS cases in which pathological size exceeded radiological size, 59% were treated by a single operation, whereas those patients in whom radiological size exceeded pathological size, 79% were treated by a single operation. These differences are highly significant (*P*⩽0.0001). The percentage of all BCS cases treated by a single successful conservation operation grouped by size difference is summarised in Figure 4. When the pathological size exceeded radiological size by >30 mm, only 14% of patients received successful single-operation BCS, whereas when radiological size was the same or greater than pathological size, 80% of patients received successful single operation BCS. This overall pattern was not influenced by the DCIS grade. In those patients who underwent eventual successful breast conservation after an initial unsuccessful conservation procedure, 54% showed a radiological size that was less than the pathological size against 71% of patients who were finally treated by mastectomy. This difference is highly significant (*P*⩽0.0001).

### Excluded graded diagnostic biopsies

Of the 304 diagnostic biopsies where data on histological grade of DCIS was available, which were excluded from the study, 126 (41%) had no further surgery, suggesting that these were in fact intended therapeutic procedures. There was a higher proportion (25%) of low-grade DCIS in this group than in the study group (7%) (*P*⩽0.0001). The data relating to this group are summarised in Table 5.

## Discussion

For a patient to have a successful single breast-conserving operation for DCIS, there is a requirement for accurate preoperative mapping of the extent of disease. The bulk of this burden falls on the radiologists, although this can be improved by using targeted core biopsies to map the full extent of the DCIS. Furthermore, inserting localisation wires at either end of the area of DCIS may assist the surgeon undertaking the resection. This is particularly useful when both benign and malignant microcalcification are known to be present.

This study examines two methods of measuring the size of DCIS – radiological and pathological. We have used the Altman–Bland plot as the statistical technique to compare the differences between radiological and pathological size assessment. For each pair of data items, the Altman–Bland plot compares the difference between the two measurements with the mean of those measurements. It therefore focuses very closely on differences. The Altman–Bland bias is the mean of these differences. A bias value of 0 indicates no difference, whereas increasing values represent greater differences. The 95% confidence intervals are also calculated. In contrast, a correlation coefficient is a measure of the degree of association between two quantities; it does not measure how closely they agree. Its use in comparing two methods that claim to measure the same parameter is inappropriate. In this context, we have restricted our use of *P*-value calculations to the comparison of radiological–pathological size differences in the subgroups analysed and radiological size estimations in the two BCS groups and the comparison of some frequencies between categorical variables.

In those patients who had successful primary BCS, there is a close agreement between radiological and pathological DCIS size, both in terms of median values and the Altman–Bland measures of bias. At all grades, the bias is in favour of radiological size, with pathological size being marginally smaller. The greatest mismatch is for low-grade disease. This is likely to be due to less calcification being associated with this grade of DCIS (Evans *et al*, 1994; de Roos *et al*, 2007); however, we urge some caution against overinterpreting the data on low-grade disease because of the relatively small numbers of cases in these subgroup analyses. Screen-detected DCIS in the United Kingdom is graded by nuclear morphology alone, as guided by the NHSBSP Reporting Guidelines (Ellis *et al*, 2005). The overall incidence of low-grade DCIS in the present series was 7%. We excluded patients with recorded coexistent atypical ductal hyperplasia and/or lobular *in situ* neoplasia because of the potential for the extent of microcalcification to exaggerate the extent of DCIS. The incidence of low-grade DCIS is lower than that reported previously from an earlier cohort of this series (11%) (Thomas *et al*, 2008) and is partly due to the exclusion of cases with coexistent atypical ductal hyperplasia/lobular *in situ* neoplasia in this study group. This low incidence may also reflect the application of stringent diagnostic criteria for grading DCIS as a result of the ongoing comprehensive training programmes required for pathologists reporting NHSBSP screen-detected cancers, as well as the recognised preponderance of detection of high-grade DCIS in the NHSBSP.

In terms of successful BCS, a small radiological overestimate of the extent of disease will guide the surgeon appropriately with the likelihood that the DCIS in question will be excised completely by one breast-conserving operation. We acknowledge that overestimation of DCIS size radiologically could lead to unnecessarily extensive surgery, but our data show that in the successful primary BCS group the radiological overestimation of DCIS size is generally small (1–3 mm) and is therefore unlikely to be detrimental in this context.

Comparison between those patients who have had one-operation BCS and primary mastectomy shows a similar bias in favour of radiological size, but allows comment to be made on the accuracy of pathologists in determining DCIS disease extent under these circumstances. Accepting that the disease process is the same in the two types of operative specimen, and that the match between the radiological and pathological size in one-operation BCS specimens is very close, it is likely that specimen handling is the major reason for the difference in estimating DCIS extent in patients having a mastectomy. This view is supported by the improvement in radiological–pathological agreement when slice radiography is used, which holds true for all tumour grades and is particularly marked for low-grade disease. Pathologists are encouraged to make use of slice radiography for cases in which microcalcification is the principal feature and these data lend support to that view (Ellis *et al*, 2005).

In those cases in which BCS was unsuccessful, median pathological size is substantially greater than radiological size for all tumour grades. This is the reverse of the situation for successful one-operation BCS procedures. This is almost certainly due to diminished microcalcification in association with the DCIS in this group of patients, even though the crude statement on the Sloane Project radiology data form ‘No calcification on mammogram’ was answered in the negative in 10% of successful primary BCS cases and in only 7% of failed primary BCS cases. There is no significant difference in the median specimen weights for the two groups, indicating that the amount of tissue removed in the two groups were comparable. However, radiological size is, unsurprisingly, significantly greater in those cases in which initial BCS was unsuccessful compared with one-operation BCS cases, at all histological grades. In the group of patients who had an unsuccessful attempt at a single surgical BCS procedure, however, the larger radiological size described here, and the increased proportion of high-grade DCIS seen (71% compared with 60% in the successful one-operation group), is not sufficiently different to be able to guide the surgeon as to whether further imaging is required to map the extent of disease. It is apparent from our data that underestimation of DCIS extent radiologically need not necessarily lead to a failed primary conservation procedure. Surgical guidelines recommend re-excision for positive margins and we have made the assumption that these cases were disease free at the margins.

The meticulous studies of Holland *et al* comparing radiological and pathological disease extent indicated that radiology systematically underestimated DCIS size and run counter to the data presented here (Holland *et al*, 1990; Holland and Hendricks, 1994). Holland's series of cases from one surgical unit is very different from the wide cross-section seen in the Sloane Project; in the former series, there were much smaller numbers (83 and 119, respectively) and the specimens were all mastectomies. In our series, this radiological underestimation of DCIS size only applies to the subset of failed primary BCS cases (about 30% of conservation cases). The remaining 70% were surgically clear with a close agreement between radiological and pathological size.

Our data from primary mastectomies show underestimation of disease extent by pathologists who are, overall, unlikely to have emulated Holland's very detailed approach. A comparable study of 109 mastectomy specimens showed no significant difference between radiological and pathological measurements of DCIS extent (Sato *et al*, 2002). A more recent study of 174 cases of DCIS showed a correlation between increasing pathological–radiological size discrepancy and the requirement for more than one operation (Chakrabarti *et al*, 2006). That study failed to show a relationship between size discrepancy and grade, although case numbers were considerably lower than reported here. Ultimately the significance of these mismatches and the relevance of margin clearances will be resolved when outcome data (recurrence and survival) emerge for the Sloane Project cohort.

Although the Sloane Project pathology data form requests details regarding the cut-up method used by pathologists, there is no opportunity for the pathologist to describe his/her measurement technique. It is much easier to measure the size of a lesion in a wide local excision specimen because the sections can either be re-assembled like a jigsaw puzzle to give an overall size or large sections can be used that allow direct measurements. Such use of large block histology has been shown to improve the agreement between radiological and pathological lesion size (Jackson *et al*, 1994), but in this series there were insufficient cases following the various exclusions to be able to confirm this finding. Alternatively, the pathologist can relate the disease to its distance from margins and then calculate the extent from the overall dimensions of the specimen (Lester *et al*, 2009). It has also been suggested that the size may be estimated by the number of blocks containing DCIS multiplied by a factor, but a recent study has cast doubt on the accuracy of this approach (Dadmanesh *et al*, 2009). Inconsistency among pathologists in measuring DCIS extent has also been previously reported (Sloane *et al*, 1999).

We have not analysed the margin status or width in this study, believing this to be much more pertinent to a future analysis of the Sloane Project data examining outcomes, particularly local recurrences, when longer follow-up is available. The overwhelming majority of patients receiving a single successful conservation operation have negative margins, as defined by the particular centre. In some cases, a positive margin will not result in further surgery – most commonly anterior or posterior margins in full-thickness procedures. It should be appreciated that exactly what defines a negative margin (‘complete excision’) differs from centre to centre and that variation will itself enable an evaluation of optimal margin clearance when outcome data become available.

Are there any developments that could lead to a reduction of re-operation rates for DCIS? The College of American Pathologists has recently issued guidelines on specimen handling for DCIS that lay down stringent standards for this area of practice (Lester *et al*, 2009). These explicit recommendations are most likely to improve pathological assessment of disease extent and reproducibility of grade of DCIS and may improve detection of invasive disease. They will however do nothing to affect accurate preoperative assessment of disease extent, unless accompanied by the rigorous application of radiological–pathological size correlation of both operative and core biopsy specimens (e.g., if multiple cores are taken from areas of microcalcification with associated pathological–radiological mapping). More detailed discussion at the multidisciplinary meeting of the extent of calcification within the DCIS (in one or multiple core biopsies) can be correlated with how well the extent of disease is likely to be represented by the mammographic appearances, particularly in intermediate and low-grade DCIS. The use of wide-bore, vacuum-assisted, needle biopsy may also improve diagnosis and assessment in difficult cases (Pijnappel *et al*, 2004).

Studies have suggested that magnification views can aid the prediction of extent of low-grade DCIS (Asjoe *et al*, 2007; Di Saverio *et al*, 2008). Our data show that the size discrepancies leading to failed primary BCS occur across all tumour grades and that, numerically, low-grade disease is uncommon (<10% of cases). The overall impact of this approach to low-grade disease alone would therefore be small. Furthermore, it has been reported that the mammographic appearances do not correlate sufficiently with histological grade to assist preoperative identification of this subset of problem cases (de Roos *et al*, 2004). Histological reporting of the grade of DCIS in the core biopsy specimen, however, may prove of assistance in highlighting cases in which size discrepancy may be greatest, as core biopsy grade of DCIS has been shown to reflect the subsequent DCIS grade seen in surgical excision (Bagnall *et al*, 2001).

Recently, attention has been focused on the value of magnetic resonance imaging (MRI) in determining disease extent of DCIS. A number of recent studies have compared the ability of MRI and mammography to detect and size DCIS lesions. The results are conflicting; a number favouring MRI (Schouten van der Velden *et al*, 2006; Kim *et al*, 2007; van Goethem *et al*, 2007), another finding the performance of MRI and mammography were similar (Sardanelli *et al*, 2008) and yet another finding that the combination of mammography and MRI was significantly better than either of the modalities alone (Santamaría *et al*, 2008).

The main limitation of this study relates to the inevitable range of practice from centre to centre. Although this is minimised by the availability of practice guidelines that have been in place in the NHSBSP for the past 20 years, there is bound to be a degree of variability. We have not attempted to review the data from individual centres at this time because the numbers of cases from each centre will be too small to analyse, but this will be possible in the future as the Sloane Project case accrual grows and that will allow differences in practice to be compared against the outcomes. To minimise the effects of such variation, we have restricted our analyses to very broad groupings of cases with large numbers in each.

## Conclusions

In a large series of screen-detected DCIS, we have shown that current approaches to preoperative imaging undersize the extent of disease in patients selected for BCS in up to 30% of cases, with the consequence of failed primary conservation surgery. Nevertheless, breast conservation can still be achieved in two-thirds of this group and by the second operation in 90% of cases.

Multidisciplinary team working is likely to have made a substantial contribution to effective management of breast cancer over the past 20 years and could be expected to have an impact on DCIS. Further improvements in preoperative assessment should include detailed discussion between surgeon, radiologist and pathologist about radiological–pathological size correlation, particularly the extent of colocation of microcalcification and DCIS. Our data suggest that such discussion should be particularly targeted at intermediate and low-grade disease.

## Figures and Tables

**Table 1:** Grade distribution of DCIS by operation type

	**DCIS grade**						
	**All grades**	**High**		**Intermediate**		**Low**	
**Type of operation**	**No. of cases**	**No. of cases**	**%**	**No. of cases**	**%**	**No. of cases**	**%**
Successful primary BCS	1430	857	60	448	31	125	9
Primary mastectomy	551	441	80	86	16	24	4
Unsuccessful primary BCS	583	411	71	147	25	25	4
Totals	2564	1709	67	681	26	174	7

Abbreviations: BCS=breast-conserving surgery; DCIS=ductal carcinoma *in situ.*

**Table 2:** Radiological and pathological size measurements for each operation type and the influence of grade and specimen slice radiography

			**DCIS grade**							
			**All**		**High**		**Intermediate**		**Low**	
**Type of operation**	**Specimen slice X-ray**	**Type of size**	**No. of cases**	**Median size (mm)**	**No. of cases**	**Median size (mm)**	**No. of cases**	**Median size (mm)**	**No. of cases**	**Median size (mm)**
Successful primary BCS	All cases	Pathological	1430	12	857	14	448	10	125	7
		Radiological	1430	14	857	15	448	12	125	9
		**Difference**		**2**		**1**		**2**		**2**
	No	Pathological	836	12	470	13	290	10	76	7
		Radiological	836	13	470	15	290	12	76	9
		**Difference**		**1**		**2**		**2**		**2**
	Yes	Pathological	455	14	305	16	108	10	42	7
		Radiological	455	15	305	18	108	13	42	9
		**Difference**		**1**		**2**		**3**		**2**
Primary mastectomy	All cases	Pathological	551	32	441	35	86	27	24	18
		Radiological	551	49	441	50	86	46	24	64
		**Difference**		**17**		**15**		**19**		**46**
	No	Pathological	168	31	127	35	34	27	7	16
		Radiological	168	48	127	50	34	46	7	48
		**Difference**		**17**		**15**		**19**		**32**
	Yes	Pathological	125	31	96	37	21	22	9	18
		Radiological	125	45	95	48	21	37	9	80
		**Difference**		**14**		**11**		**15**		**62**
Unsuccessful primary BCS	All cases	Pathological	583	22	411	24	147	19	25	27
		Radiological	583	16	411	18	147	14	25	15
		**Difference**		**−6**		**−6**		**−5**		**−12**
	No	Pathological	339	23	237	24	83	21	14	27
		Radiological	339	16	237	19	83	14	14	12
		**Difference**		**−7**		**−5**		**−7**		**−15**
	Yes	Pathological	177	20	123	24	41	17	8	22
		Radiological	177	16	123	20	41	12	8	18
		**Difference**		**−4**		**−4**		**−5**		**−4**

Abbreviations: BCS=breast-conserving surgery; DCIS=ductal carcinoma *in situ.*

Note that all differences in the unsuccessful BCS group are negative.

**Table 3:** Outcome of patients requiring further surgery for failed primary breast-conserving surgery

	**No. of further operations**				
	**1**	**2**	**3**	**Total**	**%**
Successful Conservation	**368**	**11**	**1**	**380**	**65**
Mastectomy	**169**	**30**	**4**	**203**	**35**
Total	**537**	**41**	**5**	**583**	

**Table 4:** Altman–Bland bias as a measure of agreement between radiological and pathological measurements of DCIS by operation type and the influence of grade and specimen slice radiography

		**DCIS grade**							
		**All**		**High**		**Intermediate**		**Low**	
**Type of operation**	**Specimen slice X-ray**	**No. of cases**	**Bias**	**No. of cases**	**Bias**	**No. of cases**	**Bias**	**No. of cases**	**Bias**
Successful primary BCS	All cases	1430	2.96	857	2.67	448	3.46	125	3.12
	No	836	3.36	470	3.10	290	3.70	76	3.80
	Yes	455	2.63	305	2.40	108	3.60	42	1.80
Primary mastectomy	All cases	551	13.33	441	11.35	86	18.60	24	30.70
	No	168	13.00	127	11.80	34	16.00	7	19.90
	Yes	125	13.10	95	10.60	21	13.80	9	37.70
Unsuccessful primary BCS	All cases	583	−6.46	411	−6.6	147	−5.06	25	−12.30
	No	339	−7.94	237	−7.48	88	−8.0	14	−15.60
	Yes	177	−3.06	123	−3.7	46	−1.41	8	−2.10

BCS=breast-conserving surgery; DCIS=ductal carcinoma *in situ.*

Influence of grade and specimen slice radiography. Note that all bias values in the unsuccessful primary BCS group in are negative

**Table 5:** Analysis of excluded graded diagnostic biopsies

**DCIS grade**	**All**	**High**	**Intermediate**	**Low**
Number (%)	304	114 (38)	113 (37)	77 (25)
No further surgery	126	33	46	47
Further excision to clear margins	70	29	25	16
Mastectomy^a^	46	26	14	6
Wide local excision	33	16	15	2
Multiple operations^b^	26	9	13	4
Axillary surgery only	1			1
Median Pathology size (mm)	12	17	10	8
Median radiology size (mm)	17	17	17	15

DCIS=ductal carcinoma *in situ.*

a Excludes multiple operations group (see below).

b 18 of these cases were ultimately treated by mastectomy.

## Appendix

**Membership of the Sloane Project Steering Group:**

Mr Hugh Bishop, Consultant Surgeon, Royal Bolton Hospital, Bolton (Chair), UK.

Mr Rob Carpenter, Consultant Breast Surgeon, St Bartholomew's and the London Hospitals, London, UK.

Ms Karen Clements, West Midlands Cancer Intelligence Unit, Birmingham, UK.

Dr John Dewar, Consultant Clinical Oncologist, Ninewells Hospital, Dundee, UK.

Dr Hilary Dobson, Director, West of Scotland Breast Screening Service, UK.

Professor David Dodwell, Consultant Clinical Oncologist, Leeds Teaching Hospitals, Leeds, UK.

Professor Ian Ellis, Professor of Tumour Pathology, City Hospital, Nottingham, UK.

Professor Andrew Evans, Professor of Breast Imaging, Ninewells Hospital, Dundee, UK.

Professor Andrew Hanby, Consultant Breast Pathologist, St James’ University Hospital, Leeds

Ms Olive Kearins, West Midlands Cancer Intelligence Unit, Birmingham, UK.

Dr Gill Lawrence, Director, West Midlands Cancer Intelligence Unit, Birmingham, UK.

Mr Martin Lee, Consultant Surgeon, University Hospital, Coventry, UK.

Dr Anthony Maxwell, Consultant Radiologist, Royal Bolton Hospital, Bolton, UK.

Mr Stewart Nicholson, Consultant Surgeon, York Hospitals, York, UK.

Professor Sarah Pinder, Professor of Breast Pathology, Guy's and St Thomas’ Hospitals, King's College London, London, UK.

Mr Terry Roberts, West Midlands Cancer Intelligence Unit, Birmingham, UK.

Dr Jeremy Thomas, Consultant Breast Pathologist, Western General Hospital, Edinburgh, UK.

Dr Matthew Wallis, Consultant Radiologist, Addenbrooke's Hospital, Cambridge, UK.

Mrs Margot Wheaton, Programme Manager, Warwick, Solihull and Coventry Breast Screening Service, UK.

**Participating Screening Units in the Sloane Project as per October 2009**

Avon

Barking, Havering, Brentwood and Redbridge

Barnsley

Basingstoke and District

Bedfordshire and Hertfordshire

Bolton, Bury and Rochdale

Breast Test Wales – North

Breast Test Wales – South East

Breast Test Wales – South West

Cambridge and Huntingdon

Central and East London

Chelmsford and Colchester

Chester

City, Sandwell and Walsall

Cornwall

Crewe

Doncaster

Dorset

Dudley and Wolverhampton

East Berkshire

East Scotland

East Sussex

Gateshead

Gloucestershire

Great Yarmouth and Waveney

Greater Manchester

Hereford and Worcester

Humberside

Isle of Wight

Jarvis

Leeds and Wakefield

Leicestershire

Liverpool

Macclesfield

Maidstone

Medway

Milton Keynes

Newcastle-Upon-Tyne

Norfolk and Norwich

North and East Devon

North Cumbria

North East Scotland

North Lancashire and South Cumbria

North London

North Nottinghamshire

North Staffordshire

North Yorkshire

Northampton

Nottingham

Oxford

Pennine (Bradford)

Peterborough

Portsmouth

Rotherham

Sheffield

Shropshire

South Birmingham

South Derbyshire

South Devon

South East London

South East Scotland

South Essex

South Staffordshire

South West London

South West Scotland

Southampton and Salisbury

Warrington

Warwickshire, Solihull and Coventry

West Berkshire

West Devon and East Cornwall

West Essex and Redbridge

West London

West Scotland

Western Breast Screening Unit, Northern Ireland

Wiltshire

Wirral

Wycombe
